# Auger radiopharmaceutical therapy targeting prostate-specific membrane antigen in a micrometastatic model of prostate cancer

**DOI:** 10.7150/thno.38882

**Published:** 2020-02-03

**Authors:** Colette J. Shen, Il Minn, Robert F. Hobbs, Ying Chen, Anders Josefsson, Mary Brummet, Sangeeta R. Banerjee, Cory F. Brayton, Ronnie C. Mease, Martin G. Pomper, Ana P. Kiess

**Affiliations:** 1 Department of Radiation Oncology and Molecular Radiation Sciences, Johns Hopkins University School of Medicine, Baltimore, MD; 2 Russell H. Morgan Department of Radiology and Radiological Science, Johns Hopkins University School of Medicine, Baltimore, MD; 3 Department of Molecular and Comparative Pathobiology, Johns Hopkins University School of Medicine, Baltimore, MD; 4 Current affiliation: Department of Radiation Oncology, University of North Carolina School of Medicine, Chapel Hill, NC

**Keywords:** Auger, radiopharmaceutical therapy, radionuclide therapy, PSMA, prostate cancer, micrometastatic

## Abstract

Auger radiopharmaceutical therapy is a promising strategy for micrometastatic disease given high linear energy transfer and short range in tissues, potentially limiting normal tissue toxicities. We previously demonstrated anti-tumor efficacy of a small-molecule Auger electron emitter targeting the prostate-specific membrane antigen (PSMA), 2-[3-[1-carboxy-5-(4-[^125^I]iodo-benzoylamino)-pentyl]-ureido]-pentanedioic acid), or ^125^I-DCIBzL, in a mouse xenograft model. Here, we investigated the therapeutic efficacy, long-term toxicity, and biodistribution of ^125^I-DCIBzL in a micrometastatic model of prostate cancer (PC).

**Methods**: To test the therapeutic efficacy of ^125^I-DCIBzL in micrometastatic PC, we used a murine model of human metastatic PC in which PSMA+ PC3-ML cells expressing firefly luciferase were injected intravenously in NSG mice to form micrometastatic deposits. One week later, 0, 0.37, 1.85, 3.7, 18.5, 37, or 111 MBq of ^125^I-DCIBzL was administered (intravenously). Metastatic tumor burden was assessed using bioluminescence imaging (BLI). Long-term toxicity was evaluated via serial weights and urinalysis of non-tumor-bearing mice over a 12-month period, as well as final necropsy.

**Results**: In the micrometastatic PC model, activities of 18.5 MBq ^125^I-DCIBzL and above significantly delayed development of detectable metastatic disease by BLI and prolonged survival in mice. Gross metastases were detectable in control mice and those treated with 0.37-3.7 MBq ^125^I-DCIBzL at a median of 2 weeks post-treatment, versus 4 weeks for those treated with 18.5-111 MBq ^125^I-DCIBzL (*P*<0.0001 by log-rank test). Similarly, treatment with ≥18.5 MBq ^125^I-DCIBzL yielded a median survival of 11 weeks, compared with 6 weeks for control mice (*P*<0.0001). At 12 months, there was no appreciable toxicity via weight, urinalysis, or necropsy evaluation in mice treated with any activity of ^125^I-DCIBzL, which represents markedly less toxicity than the analogous PSMA-targeted α-particle emitter. Macro-to-microscale dosimetry modeling demonstrated lower absorbed dose in renal cell nuclei versus tumor cell nuclei due to lower levels of drug uptake and cellular internalization in combination with the short range of Auger emissions.

**Conclusion**: PSMA-targeted radiopharmaceutical therapy with the Auger emitter ^125^I-DCIBzL significantly delayed development of detectable metastatic disease and improved survival in a micrometastatic model of PC, with no long-term toxicities noted at 12 months, suggesting a favorable therapeutic ratio for treatment of micrometastatic PC.

## Introduction

Limited therapeutic options exist for men with castration-resistant metastatic prostate cancer (PC). While several systemic agents have recently shown promise, including enzalutamide, abiraterone, docetaxel, and cabazitaxel, the absolute survival benefits have been small, on the order of 2-5 months 1-3. Success with the α-particle emitter ^223^RaCl_2_ for patients with metastatic PC demonstrates the potential for systemic radiopharmaceutical therapy (RPT) to improve survival with limited toxicity 4. However, this therapy only prolonged survival by 3 months and is limited to treatment of bone metastases.

Recent efforts have turned toward targeting prostate-specific membrane antigen (PSMA), a cell surface antigen expressed >1,000-fold in nearly all PC relative to normal prostate tissue 5,6, which allows targeting of both bone and soft tissue metastases 7. Clinical studies evaluating PSMA-targeted agents for therapeutic purposes have utilized primarily β-particle-emitting radionuclides conjugated either to PSMA-targeting small molecules 8,9 or anti-PSMA antibodies 10,11. While an anti-PSMA antibody β-emitter was associated with hematologic toxicity 10, small molecule β-emitters targeting PSMA (*e.g.* ^177^Lu-PSMA617) have shown anti-tumor efficacy with minimal hematologic toxicity 9,12. PSMA-targeted α-particle and Auger emitters have potential for increased therapeutic ratio due to shorter range and higher linear energy transfer (LET) than β-particle emitters 13. Early clinical studies of the α-particle emitter ^225^Ac-PSMA617 showed higher antitumor efficacy and lower bone marrow toxicity compared to the β-particle emitter ^177^Lu-PSMA617, although dose-limiting xerostomia was noted 14,15.

Our group has recently demonstrated that a small molecule α-particle emitter with ^211^At targeting PSMA, (2*S*)-2-(3-(1-carboxy-5-(4-^211^At-astatobenzamido) pentyl)ureido)-pentanedioic acid (^211^At-PSMA-6), significantly improved survival in mice bearing PC micrometastases, but at the expense of lethal late nephrotoxicity 16. We have previously shown that the corresponding PSMA-targeting small molecule that emits Auger electrons, 2-[3-[1-carboxy-5-(4-[^125^I]iodo-benzoylamino)-pentyl]-ureido]-pentanedioic acid), or ^125^I-DCIBzL, has anti-tumor efficacy in mice bearing PC xenografts 17,18. ^125^I, a radioisotope of iodine with a longer half-life of 59 days that decays via emission of Auger electrons and low-energy photons, is one of the most characterized and studied Auger electron emitters both *in vitro* and *in vivo* 19,20.

Auger emitters have even shorter range than α-particle emitters, making them well suited for treatment of micrometastases with sparing of nearby normal tissues 13,19,21. A few other studies have demonstrated antitumor efficacy of systemic Auger RPT in pre-clinical models, including metastatic melanoma 22 and glioblastoma 23. Several phase I clinical trials have evaluated Auger RPT in humans. Patients with neuroendocrine tumors were treated with ^111^In-DTPA-octreotide at doses of 20-160 GBq, with therapeutic effect seen in about 50% of patients; however, a MTD of 100 GBq was established due to development of myelodysplastic syndrome or leukemia 24. In another trial using this compound, patients received two treatments of 6.6 GBq each, which were well-tolerated but only resulted in partial response in a small percent of patients 25, possibly related to only modest uptake of this compound into cell nuclei 26. More recently, ^111^In-DTPA-hEGF has been evaluated in a Phase I trial of patients with epidermal growth factor receptor-positive metastatic breast cancer at activities up to 2.3 GBq, which were well tolerated (MTD not reached) but also did not result in objective antitumor responses 27.

In this study, we hypothesized that the PSMA-targeting Auger electron emitter ^125^I-DCIBzL would have less toxicity and higher therapeutic ratio for treatment of micrometastases than the corresponding α-particle emitter (^211^At-PSMA-6) due to its very short range (<10μm). We sought to evaluate the anti-tumor efficacy of ^125^I-DCIBzL in a micrometastatic model of PC in mice. We also assessed long-term toxicity and conducted further dosimetry modeling of ^125^I-DCIBzL.

## Materials and Methods

### Chemistry

Methods have been previously described for the synthesis of ^125^I-DCIBzL 17. This compound has demonstrated very high PSMA binding affinity with *K*_i_ = 0.01 nM. For this study, the specific radioactivity (molar activity) for each synthesis was > 1,200 Ci/mmol (44.4 GBq/μmol).

### Cell lines and culture conditions

The androgen-independent PC3 cell line was originally derived from a human castration-resistant PC bone metastasis. The isogenic human PC3 cell lines PIP (PSMA-positive [PSMA+]) and flu (PSMA-negative [PSMA-]) were obtained from Dr. Warren Heston (Cleveland Clinic) and maintained as described 28. The PC3-ML cell line was isolated as a subline of PC3 that preferentially metastasizes to lumbar vertebrae 29. PC3-ML-Luc (stable transfectants expressing firefly luciferase) were provided by Dr. Mauricio Reginato (Drexel University, Philadelphia, PA). PSMA-expressing PC3-ML-Luc cells (PC3-ML-Luc-PSMA) were generated via lentiviral infection as previously described 16. These were cultured in DMEM (Cellgro) supplemented with 10% (vol/vol) FBS and 1% (vol/vol) antibiotic solution (Sigma-Aldrich) and incubated at 37°C, 5% CO_2_.

### *In vivo* studies

Please refer to Table S1 for experimental conditions for the *in vivo* studies described below.

#### Antitumor efficacy in micrometastatic model

All experimental procedures involving animals were approved by the Johns Hopkins Animal Care and Use Committee, and the program is accredited by AAALAC International. Four- to 6-wk-old NSG (NOD.Cg-Prkdc^scid^IL2rg^tm1Wjl^/SzJ) mice (Animal Resources Core, Johns Hopkins) were injected intravenously with 1x10^6^ PC3-ML-Luc-PSMA cells to form micrometastatic deposits. One week later, mice (n=5/group) were injected intravenously with 0, 0.37, 1.85, 3.7, 18.5, 37, or 111 MBq of ^125^I-DCIBzL. Metastatic tumor progression was monitored by *in vivo* bioluminescence imaging (BLI) and survival. In this model, mice develop metastases within the liver, kidneys, and bone 16. BLI was performed using the IVIS Spectrum (Perkin-Elmer), with imaging 10 minutes after Luciferin injection (intraperitoneal, 200 uL of 15mg/mL solution). Mice were sacrificed if they lost >20% body weight or had signs of discomfort (immobility, fur ruffling). Survival probability was characterized using Kaplan-Meier curves, and comparison was performed using the log-rank test.

#### Long-term toxicity evaluation and necropsy

Non-tumor-bearing 6-week-old CD1 mice (Crl:CD1(ICR), Charles River), n=5/group, weighing 25-30 g received intravenous injections of 0, 0.37, 1.85, 3.7, 18.5, 37, or 111 MBq of ^125^I-DCIBzL and then were weighed and inspected twice per week for at least 12 months. Urinalysis evaluating specific gravity and urine protein levels via dipstick (Chemstrip Test Strips, Roche Diagnostics) was performed monthly for each animal. Terminal clinical and anatomic pathology was done in the Johns Hopkins Phenotyping and Comparative Pathology Core. Assessments included serum metabolic panel, blood counts, and complete necropsy with histopathology of more than 30 tissues as described previously 30.

#### Tumor and normal tissue biodistribution for long-term dosimetry

For long-term studies evaluating normal tissue biodistribution, non-tumor-bearing CD1 mice (Charles River, n=4-5/group) weighing 40-50 g were injected intravenously with 18.5 MBq of ^125^I-DCIBzL and sacrificed at 2 (n=5), 4 (n=4), 6 (n=4), 8 (n=4), 10 (n=4), and 12 (n=5) weeks. For studies evaluating biodistribution in tumor-bearing mice, NSG mice (Animal Resources core, Johns Hopkins, n=5/group) weighing 20-25 g were injected subcutaneously in the right and left flank areas with 1x10^6^ PSMA+ PC3 PIP and 1x10^6^ PSMA- PC3 flu cells, respectively. One week later, mice were injected intravenously with 18.5 MBq of ^125^I-DCIBzL and sacrificed at 1 hr, 24 hrs, 48 hrs, 72 hrs, 1 week, 2 weeks, and 3 weeks (all n=5) following injection of the compound. For both studies, the following tissues/organs were harvested and weighed, and % injected dose [ID]/g was measured using a 1282 Compugamma γ-counter (Pharmacia): blood, salivary gland, lung, heart, liver, kidney, bladder, stomach, small intestine, large intestine, pancreas, spleen, fat, muscle, and PIP/flu tumors (if applicable). Comparison between tumor and kidney uptake was performed using Student's t-test.

### Dosimetry

The organ biological data (% ID/g) from both the tumor-bearing and non-tumor bearing long-term studies were decay-corrected to the time of injection to obtain the activity in the different organs as a function of time; these data were plotted and fit with hybrid trapezoid-exponential time-activity curves. As the long-term data were well fit to an exponential curve but the earlier time points varied depending on the tissue (e.g. early uptake for the PIP tumor, rapid early clearance for the kidneys and salivary glands), trapezoidal fitting was used for the earlier time points until the remaining time point data were well fit (R^2^ > 0.9) by a mono-exponential. The electron and photon energy per nuclear transition were taken from ICRP 107: 19.2 keV for the electron and 42.8 keV for the photon emissions 31. The organ absorbed dose from the two different emission types were calculated and tabulated separately as the range of the different emissions implies different absorbed fractions; moreover, the Auger electrons are expected to have a different relative biological effectiveness (RBE) than the photons. The absorbed fraction at the organ level of the Auger electrons was assumed to be 1. The absorbed fractions for the photons were obtained for the murine kidney by GEANT4 Monte Carlo (MC) simulation using the MIRD Pamphlet #19 kidney model scaled to the mouse (mass = 298 mg) 32. Similarly, the photon absorbed fraction for tumors was derived using the same GEANT4 MC simulation for a range of 20 spheres of unit density (0.001 g - 100 g) and fitting the absorbed fractions as a function of mass using a power law function:


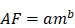
 Eq. 1

where *AF* is the absorbed fraction value in [%], *m* is the mass of the sphere in [g], *a* and *b* are fit parameters.

A potential complication for organ dosimetry with short-ranged isotopes is that the localization of activity uptake may result in a non-uniform distribution of dose such that the average organ absorbed dose may not accurately reflect observed toxicity. Small scale modeling has been used to reconcile localized activity and dose distributions with organ absorbed doses. Specifically, a nephron model of the kidneys for α-particle therapy has been published and was used as the basis for the small scale Auger dosimetry 33. This compartmental model was complemented with more cellular-level simulations as the range of Auger electrons (<10 µm) is even shorter than α-particles (50-100 µm); a tubule wall thickness of 10 µm was used and a nuclear diameter of 5 µm. Iodine-125 Auger decays were simulated in the geometry of the published nephron model using GEANT4 MC simulations, both for emissions originating on the surface of the tubules or in the cytoplasm. The energy was tabulated both at the cellular and sub-cellular (nuclear) level. Results were validated using the MIRDCell 2.0 software and comparing simple cell-sized spheres.

Cellular-level dosimetry for the PIP tumors had been performed in a previous publication using cellular diameter of 26 µm, nuclear diameter of 18 µm, and cellular distribution of 15% peri-nuclear, 35% on the cellular membrane and 50% in the cytoplasm 18. The same parameters were used with the current bio-kinetic data for more accurate tumor dosimetry.

## Results

### Antitumor efficacy in micrometastatic model

**Figure 1:**
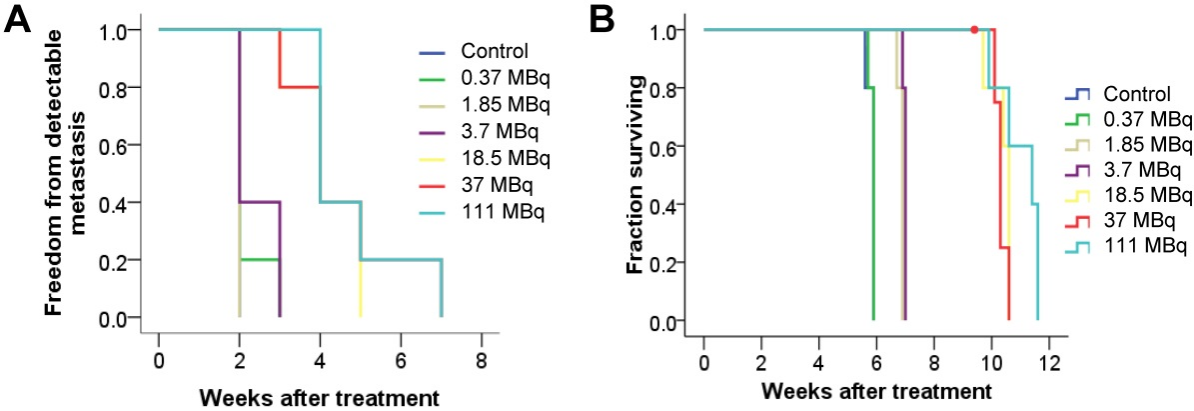
^125^I-DCIBzL treatment resulted in PC growth delay and prolonged survival in a micrometastatic mouse model. Kaplan-Meier curves show freedom from detectable metastasis via BLI (A) and overall survival (B) in mice treated with increasing doses of ^125^I-DCIBzL versus control (n=5/group). Treatment with 18.5 MBq and higher resulted in delay of metastasis growth and prolonged survival. *P*<0.0001 between control, 0.37, 1.85, and 3.7 MBq versus 18.5, 37, and 111 MBq of ^125^I-DCIBzL via log-rank test.

To evaluate antitumor efficacy in a micrometastatic PC model, we evaluated time to detectable metastases as well as overall survival in mice bearing PC micrometastases treated with increasing activities of ^125^I-DCIBzL. Median time to detectable metastases by BLI was 2 weeks for untreated mice and those receiving 0.37, 1.85, and 3.7 MBq of ^125^I-DCIBzL, compared to 4 weeks for those receiving 18.5, 37, and 111 MBq (Figure 1A, *P*<0.0001 by log-rank test; Figure S1). Median survival was 6 weeks after injection of cells for untreated mice and those treated with 0.37 MBq, 7 weeks for those treated with 1.85 and 3.7 MBq, and 10-11 weeks for mice treated with 18.5, 37, and 111 MBq (Figure 1B, *P*<0.0001; Figure S1). There was no significant difference in survival between the 18.5, 37, and 111 MBq groups.

### *In vivo* toxicity and maximum tolerated dose

**Figure 2:**
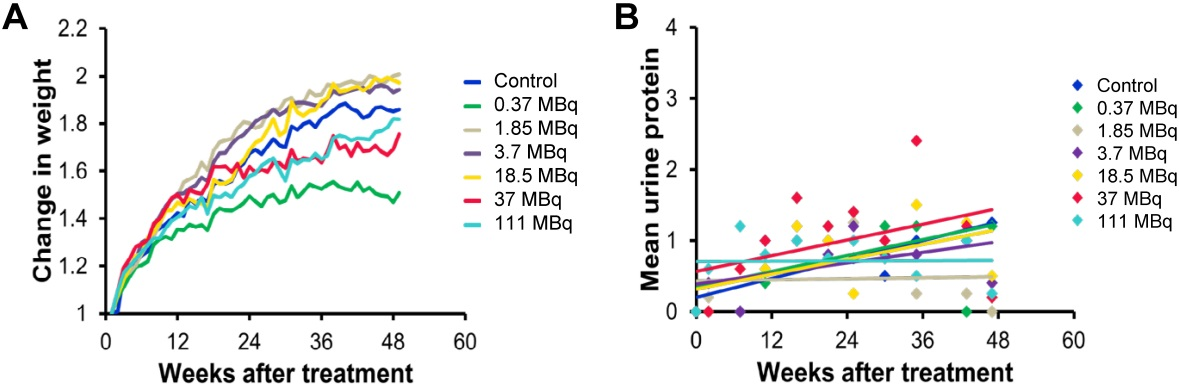
^125^I-DCIBzL treatment resulted in no long-term *in vivo* toxicity as measured by changes in body weight (A) and urine protein levels (B). Non-tumor-bearing mice were treated with increasing doses of ^125^I-DCIBzL versus control (n=5/group), and no consistent changes in weight or urine protein levels were noted in treated mice compared to controls over 1 year.

**Figure 3:**
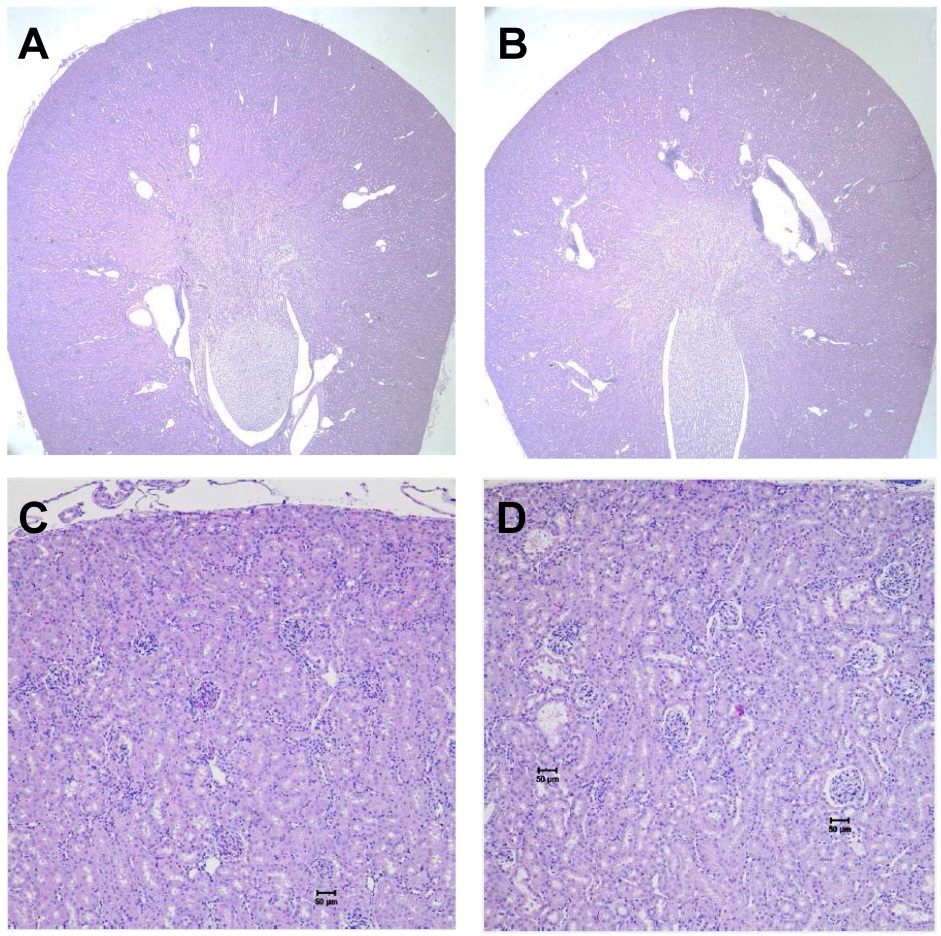
No kidney toxicity was noted 12 months after treatment with ^125^I-DCIBzL. Renal histopathology from untreated mice (A and C) and mice treated with 111 MBq of ^125^I-DCIBzL (B and D) showed renal tubule hypertrophy with occasional atypia that was modest and multifocal in the treated mice, which were within acceptable normal range for mice of this age and size. A, B: 2x; C, D: 10x. Scale bars = 50 µm.

We evaluated long-term toxicity of activities of ^125^I-DCIBzL up to 111 MBq in CD-1 mice over 12 months by blood count, serum chemistry, and histopathology of more than 30 tissues. No mouse treated with any activity of ^125^I-DCIBzL died from toxicity during this time, and there were also no consistent changes in body weight or urine protein levels (Figure 2) and no evidence of late nephropathy on necropsy (as detailed below) compared to the untreated group. Thus, the maximum tolerated dose (MTD) was not reached. These results contrast with a previous study of a similar PSMA-targeting small molecule with α-particle emitter ^211^At, which had a MTD of 37 kBq with late radiation nephropathy as the dose-limiting toxicity, as described previously 16. In this study, mice sacrificed at 12 months after receiving the highest activities of ^125^I-DCIBzL had mild renal tubule epithelial hypertrophic changes with occasional atypia (Figure 3). In all groups, renal tubule degenerative and regenerative changes, as well as mild glomerular changes, were modest and potentially normal in CD1 mice of this size and age. The average size of the kidneys in mice treated with 111 MBq of ^125^I-DCIBzL was 0.50 g compared with 0.47 g for controls, average blood urea nitrogen was 14.5 mg/dL (control, 17 mg/dL), and average creatinine was 0.4 mg/dL (control, 0.3 mg/dL). Salivary glands, which also express PSMA, had only mild changes in submandibular glands of both control and treated mice. Mild inflammatory changes were noted in lacrimal glands of treated mice. Long-term hematologic toxicity was not noted in routine histopathology of spleen, bone marrow, and lymph nodes in treated mice, and average hemoglobin at necropsy was 12.8 g/dL (control, 13.1 g/dL).

### Tumor and normal tissue biodistribution for dosimetry

**Figure 4:**
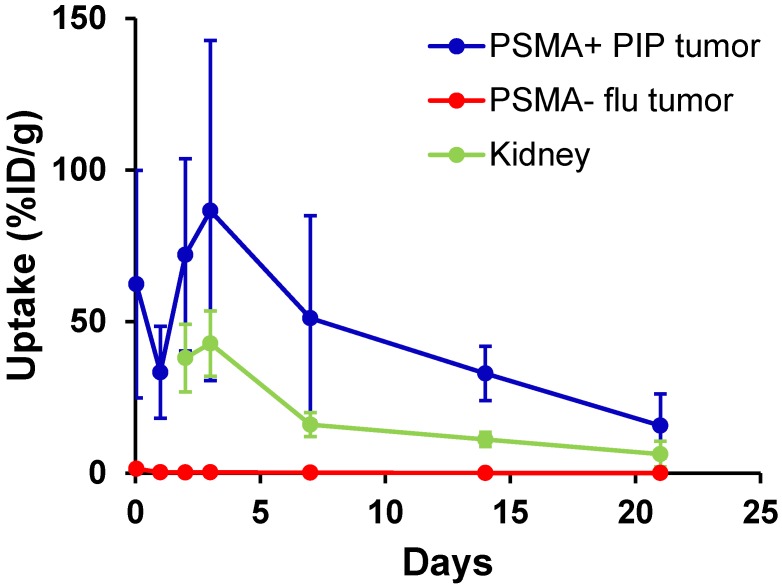
Biodistribution studies of ^125^I-DCIBzL (18.5 MBq) in mice bearing PSMA+ PC3 PIP and PSMA- PC3 flu tumors show increased uptake in PSMA+ PIP tumors compared to kidneys and PSMA- flu tumors over 3 weeks. The PIP tumor:kidney ratio remained at 2-3:1 during this period. Note that kidney uptake at 1 hr and 24 hrs was too high to be evaluated by γ-counter. Values represent mean ± standard deviation. *P*<0.05 between PIP tumor and kidney at 2 weeks via t-test, non-significant at other time points.

We evaluated long-term tumor and normal tissue biodistribution of ^125^I-DCIBzL in mice bearing PSMA+ PC3 PIP and PSMA- PC3 flu tumors (Figure 4 and Table S2). Over a 3 week period, PSMA+ PIP tumors exhibited increased uptake compared to kidneys and PSMA- flu tumors, and while uptake in both PIP tumor and kidneys decreased over this time, the PIP tumor:kidney ratio remained at 2-3:1 (*P*<0.05 at 2 week time point by t-test, non-significant at other time points). PSMA- PC3 flu tumor uptake was minimal (max 1.4 %ID/g). We also evaluated even longer-term normal tissue biodistribution of ^125^I-DCIBzL in non-tumor-bearing mice, up to 12 weeks following compound injection. Kidney uptake considerably decreased over this time, from 12.8 %ID/g at 2 weeks to 0.2 %ID/g at 12 weeks (Table S3). Further, limited thyroid and stomach uptake suggests chemical stability of ^125^I-DCIBzL with limited dehalogenation.

### Dosimetry

The average absorbed dose coefficients to kidneys for the tumor-bearing and non-tumor bearing mice from the Auger electrons were 0.99 Gy/MBq and 0.71 Gy/MBq, respectively. The slightly higher value from the tumor-bearing mice can be attributed to higher %/ID values at the earlier time points (48 and 72 h). The absorbed fraction of photons in kidneys from MC simulation was ~4%, such that the average absorbed dose coefficients to kidneys from the photons were 0.088 Gy/MBq and 0.063 Gy/MBq for the tumor-bearing and non-tumor bearing mice, respectively. For typical murine kidneys of mass = 300 mg, this translates as a range of 80-110 Gy of average absorbed dose to the kidneys for an injected activity of 111 MBq from the electrons and a range of 5-7 Gy from photons. The average absorbed dose coefficient to the salivary glands was 12 mGy/MBq, which translates to an average absorbed dose of 1.3 Gy from electrons for an injected activity of 111 MBq.

The tumor average absorbed dose coefficients from the Auger emissions were 2.6 Gy/MBq and 8.1 mGy/MBq for the PIP tumors and the flu tumors, respectively. This translates to an average absorbed dose of 290 Gy and 0.9 Gy from an injected activity of 111 MBq ^125^I-DCIBzL for the PIP and flu tumors from electrons, respectively. The parameters to the power law fit for the tumor photon S values were a = 7.2 %/g and b = 0.254 with a fitness coefficient of R^2^ = 0.982. Consequently, the average photon absorbed dose contributions to the tumors from an injected activity of 111 MBq ^125^I-DCIBzL were ~8 Gy for a PIP tumor of mass 0.001 g and ~46 Gy for a PIP tumor of mass 1 g.

Prior histopathology studies demonstrate PSMA expression on the apical surface of proximal tubule epithelial cells but not in the perinuclear area 5,34. Monte Carlo results show that, if PSMA is not internalized in these cells, only 0.8% of energy emitted by ^125^I decay on the surface of the tubule will be deposited in the nucleus resulting in DNA damage, while the remainder is deposited in the cytoplasm (34%) or in the tubule lumen. Assuming a fractional occupancy for the proximal tubule cells of 43% in the kidney and surface binding 33, the average absorbed dose to proximal tubule nuclei could be as low as 2 Gy from the Auger electrons (for an injected activity of 111 MBq), consistent with the low toxicity observed in spite of the high average absorbed dose to the kidney. Due to the very short range of the Auger electrons, the Auger contribution to the absorbed dose in the glomerular cell nuclei can be considered to be negligible, and the glomerular absorbed dose is effectively equal to the dose from the photon contribution.

The average absorbed dose coefficient to the salivary glands was relatively low (12 mGy/MBq), such that even if all the activity were concentrated in the PSMA-expressing striated ducts, GEANT4 MC simulations show that the average absorbed dose to the striated ducts would only be ~26 Gy from an injected activity of 111 MBq (~20 times greater than the average organ absorbed dose for salivary glands). Data are lacking concerning the cellular level of activity localization within the salivary glands, and thus cellular level dosimetry was not performed.

Small-scale simulations for the PIP tumors show that 38% of peri-nuclear decay energy is deposited in the nucleus, as compared to 5% from cell membrane activity and 8% from cytoplasmic activity, where the nuclear volume makes up 33% of the cellular volume. This translates to an average nuclear absorbed dose of 35% of the tumor, or 100 Gy for an injected activity of 111 MBq.

## Discussion

In this study, we have evaluated anti-tumor efficacy, long-term toxicity, and dosimetry of ^125^I-DCIBzL, an Auger electron-emitting small molecule RPT agent targeting PSMA. In an *in vivo* micrometastatic PC model, this compound significantly delayed time to detectable metastases and improved survival nearly two-fold (from a median of 6 weeks in untreated animals to 11 weeks in mice treated with at least 18.5 MBq ^125^I-DCIBzL; *P*<0.0001 by log-rank test). Importantly, no acute or long-term toxicity was noted for up to 12 months, even at the highest activity level. The lack of observed renal toxicity with ^125^I-DCIBzL corresponds with microdosimetry modeling demonstrating very low absorbed dose in renal cell nuclei when compared to tumor cell nuclei, due to lower levels of drug uptake and cellular internalization in combination with the very short range of Auger emissions (<10 μm). Modeling suggests that, in renal proximal tubules, the vast majority of deposited energy never reaches the renal cell nuclei (only 2 Gy for an injected activity of 111 MBq). This is consistent with apical surface rather than cytoplasmic localization of PSMA in immunohistochemistry studies in renal proximal tubules 5,34. Conversely, tumor cells internalize a substantial fraction of the radiopharmaceutical to the perinuclear area, such that ~50 times higher dose reaches the nucleus (100 Gy for an injected activity of 111 MBq) 18. Some studies have suggested that Auger electrons may cause cytotoxicity via damage to the plasma membrane or intracellular proteins, but the lack of renal toxicity in our study is most consistent with a requirement for nuclear proximity and direct DNA damage at least in normal renal tissues 21,35. Another potential contributor to the difference in absorbed dose in tumor versus renal cell nuclei is the observation that PSMA is localized to a membrane compartment near mitotic spindle poles 36, and less frequent cell division of renal proximal tubule cells compared to tumor cells may thus limit their exposure to a short-range Auger electron emitter targeting PSMA. Long-term biodistribution studies of ^125^I-DCIBzL in tumor-bearing mice showed the tumor:kidney uptake was consistently 2-3:1 from 48 hours through 3 weeks following injection of the compound. We note that there appears to be no additional therapeutic effect above 18.5 MBq, which is likely due to target saturation, and thus higher activities may not be beneficial but repeat treatment can be considered.

A few caveats exist regarding the dosimetry: (1) As with all models, the value depends on the input data. Localization of ^125^I-DCIBzL on the normal organ cell surface has not been observed directly for the kidneys or the salivary glands. However, immunohistochemistry studies have demonstrated expression of PSMA on the apical surface of the proximal tubule epithelial cells without perinuclear localization 5,34. Furthermore, the assumption of minimal internalization of ^125^I-DCIBzL in normal tissues is consistent with the lack of toxicity. (2) Calculations are based on single cell and nuclear sizes as well as a nucleus situated in the geometric center of the cells. (3) The long half-life of ^125^I reduces the effectiveness of the treatment 37. Traditionally, the biologically effective dose (BED) is used as a measure of the dose rate effect; in this paradigm, the BED is almost certainly nearly identical to the absorbed dose for the values calculated here. However, the standard BED formalism does not take into account tumor repopulation, which in the context of ^125^I therapy should be considered for correlation with tumor outcome. (4) The photon absorbed dose contribution has been applied only as self-dose. In addition, there will be photon absorbed dose contribution to all tissues from external sources (other organs and tissues). (5) The tubule absorbed dose value can be expected to vary substantially, as it is unlikely that the entirety of the proximal tubules have a similar amount of uptake. (6) Dosimetry calculations were based on biodistribution studies in two different mouse strains—CD1 mice for long-term normal tissue biodistribution in non-tumor-bearing mice and NSG mice for tumor and normal tissue biodistribution in tumor-bearing mice (due to engraftment of tumor only in NSG mice). The pharmacokinetics of ^125^I-DCIBzL may differ between mouse strains, and we also note that the higher weight of the CD1 mice could also impact biodistribution results. In addition, dosimetry calculations for tumor were based on PC3-PIP xenografts, which have higher than endogenous-level expression of PSMA, and thus may overestimate the therapeutic index based on dosimetry. We also note that for tumor uptake results, the tumor size initially decreased at early time points and then increased to above the initial size at week 3, which may contribute to the “peak” in %ID/g at the early time points.

The results of this study confirm our hypothesis that the PSMA-targeted Auger emitter ^125^I-DCIBzL demonstrates a higher therapeutic ratio than the analogous α-particle emitter ^211^At-PSMA-6 in an *in vivo* murine model of micrometastatic PC 16,18. We had previously observed anti-tumor efficacy but dose-limiting late nephrotoxicity with ^211^At-PSMA-6 16. The MTD of ^211^At-PSMA-6 was only 37 kBq in CD1 mice, whereas the MTD of ^125^I-DCIBzL was not reached and was at least 111 MBq in CD1 mice. In the PSMA-positive micrometastatic PC model, a similar 2-fold increase in median survival was observed with 740 kBq of ^211^At-PSMA-6 and with 18.5 MBq of ^125^I-DCIBzL; however, this represented 20 times the MTD for ^211^At-PSMA-6 compared with less than 1/6^th^ of the MTD for ^125^I-DCIBzL. In addition to minimal nephrotoxicity, ^125^I-DCIBzL was associated with minimal salivary gland toxicity and no hematologic toxicity on necropsy or blood counts. We do note that salivary uptake of PSMA-targeted therapies may be different between mouse and human salivary glands and thus that data from mouse studies may not be directly applicable to humans; however, human salivary uptake and toxicity also appear to vary depending on the agent used 5,38,39.

While identifying appropriate clinical doses for Auger RPT that optimize the therapeutic ratio will require extensive study for each compound, the results of early clinical studies of other Auger RPT 24,25,27 suggest that the activities of Auger emitter used in this pre-clinical mouse model (therapeutic effect seen at 18.5 MBq) are potentially relevant and translatable to human studies when scaled accordingly. However, major radiation safety precautions will be required for clinical translation of ^125^I-DCIBzL given the very high required activities, long half-life of 59 days requiring long-term radioactive waste storage, low-energy photon emissions, and risks of free ^125^I. Auger emitters with shorter half-lives, including ^111^In and ^123^I, can be considered but emit fewer electrons per decay. We also note that the contribution of low-energy photons to both individual organ and whole-body absorbed dose will be higher in humans compared to mice, both from scaling by weight and from the higher probability that emitted photons will deposit their energy within the human body as compared with a mouse. These factors, in addition to the high cost of manufacturing ^125^I-DCIBzL at the activities needed for human studies, will need to be considered when translating Auger emitter therapies to the clinical setting.

A concern regarding Auger emitter therapy is that the compound needs to be in close proximity to the nucleus given the short range of Auger electrons, and our previous work confirmed specific uptake and perinuclear/intracellular localization of the PSMA-targeting small molecule in PSMA-expressing PC cells 18. Furthermore, our current dosimetry results suggest that this short range provides a larger differential dose (~50x higher) to tumor cell nuclei compared to normal tissue (kidney) nuclei. Our results in a micrometastatic PC model demonstrate significant survival benefit and an even higher therapeutic ratio than the corresponding PSMA-targeting α-particle emitter ^211^At-PSMA-6 16, confirming the potential for Auger emitter therapy in treatment of micrometastatic disease.

Another concern with Auger emitter therapy that its efficacy may be limited to micrometastatic disease, since the targeting compound may need be internalized by every cell, and tumor penetration and uniformity of uptake are typically limited with larger tumors. However, this is somewhat mitigated by the small size of the targeting molecule and by the non-negligible photon dose component, which increases proportionately as a function of tumor size. Indeed, our earlier results with PC flank xenografts (macrometastases) suggest that there is still considerable efficacy in the macroscopic disease setting. There is also the possibility of bystander effect contributing to tumor cell kill, which may be investigated in future studies. Thus, in addition to treatment of micrometastatic prostate cancer, PSMA-targeting Auger-based RPT can potentially be used to treat macrometastatic disease when normal tissue toxicity must be minimized. Its role can also be expanded by considering combination therapies, for example with β-particle emitters, external beam radiotherapy, or repeat dosing to target additional cells.

## Conclusion

PSMA-targeted RPT with the Auger emitter ^125^I-DCIBzL significantly delayed development of detectable metastatic disease and improved survival in a micrometastatic model of PC. Importantly, no long-term toxicities were noted for up to 12 months, indicating a high therapeutic ratio when compared to other PSMA-targeting RPT agents. These results suggest that ^125^I-DCIBzL should be considered for further development for treatment of metastatic PC.

## Supplementary Material

Supplementary figure and tables.

## Abbreviations

BED: biologically effective dose
BLI: bioluminescence imaging
Bq: becquerel
Gy: gray
^125^I- DCIBzL: 2-[3-[1-carboxy-5-(4-[^125^I]iodo-benzoylamino )-pentyl]-ureido]-pentanedioic acid)
ID: injected dose
LET: linear energy transfer
MC: Monte Carlo
MTD: maximum tolerated dose
PC: prostate cancer
PSA: prostate-specific antigen
PSMA: prostate-specific membrane antigen
RBE: relative biological effectiveness
RPT: radiopharmaceutical therapy
