# Supplementary material for: Auger radiopharmaceutical therapy targeting prostate-specific membrane antigen in a micrometastatic model of prostate cancer
Source: Theranostics. 2020 Feb 3;10(7):2888–96. doi: 10.7150/thno.38882 (PMC7053212; doi:10.7150/thno.38882)
Supplement: Supplementary file 1 — Supplementary figure and tables. [file thnov10p2888s1.pdf]

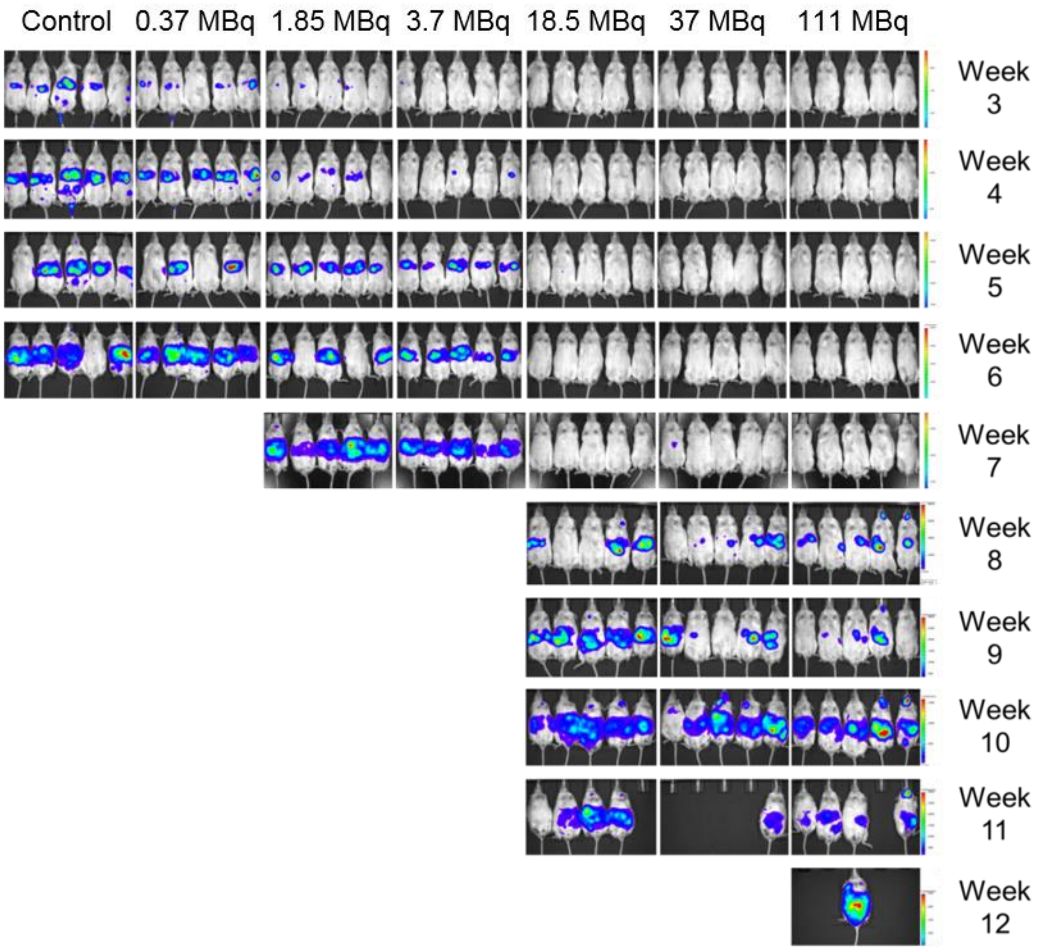

Figure S1: Mice injected with PC3-ML-Luc-PSMA cells via tail vein to establish micrometastases were treated 1 week later (n=5/group) with 0, 0.37, 1.85, 3.7, 18.5, 37, or 111 MBq of ^{125}I-DCIBzL. Metastatic tumor progression was monitored by *in vivo* bioluminescence imaging beginning 2 weeks after treatment.

Table S1. In vivo experimental conditions

Experiment	Length of study	Mouse strain	Cell type
Anti-tumor efficacy of ^{125}I-DCIBzL, micrometastatic prostate cancer model	12 weeks	NSG	PC3-ML-Luc-PSMA
Toxicity of ^{125}I-DCIBzL in non-tumor-bearing mice	12 months	CD1	N/A
Biodistribution of ^{125}I-DCIBzL in tumor-bearing mice	3 weeks	NSG	PSMA+ PC3 PIP, PSMA- PC3 flu
Biodistribution of ^{125}I-DCIBzL in non-tumor-bearing mice	12 weeks	CD1	N/A

Abbreviations: NSG: NOD.Cg-Prkdc^{scid}IL2rg^{tm1Wjl}/SzJ; PSMA: prostate-specific membrane antigen

Table S2: Biodistribution of ^{125}I-DCIBzL in tumor-bearing mice

	**% Injected Dose/gram**													
	1 hr		24 hrs		48 hrs		72 hrs		1 week		2 weeks		3 weeks	
	Mean	SD	Mean	SD	Mean	SD	Mean	SD	Mean	SD	Mean	SD	Mean	SD
Blood	1.24	0.45	0.06	0.01	0.03	0.01	0.03	0.00	0.01	0.00	0.01	0.00	0.00	0.00
Salivary gland	2.21	0.56	0.53	0.08	0.27	0.06	0.24	0.05	0.12	0.03	0.10	0.03	0.05	0.04
Thyroid	0.84	0.40	0.23	0.12	0.31	0.26	0.17	0.13	0.17	0.16	0.09	0.05	0.04	0.03
Lung	3.53	0.86	0.62	0.05	0.33	0.04	0.28	0.03	0.14	0.03	0.11	0.02	0.05	0.03
Heart	1.36	0.38	0.19	0.04	0.13	0.10	0.08	0.04	0.03	0.01	0.03	0.01	0.01	0.01
Liver	14.04	1.64	0.69	0.15	0.27	0.03	0.25	0.04	0.09	0.02	0.05	0.01	0.02	0.02
Kidney*	n/a	n/a	n/a	n/a	37.95	11.15	42.74	10.78	15.97	3.98	11.08	2.38	6.23	4.19
Bladder	3.03	0.50	2.08	0.76	0.72	0.56	0.53	0.39	0.17	0.08	0.08	0.01	0.03	0.02
Stomach	1.22	0.32	0.20	0.09	0.09	0.01	0.12	0.05	0.05	0.01	0.03	0.01	0.01	0.01
Pancreas	1.57	0.42	0.33	0.10	0.19	0.11	0.13	0.03	0.07	0.03	0.05	0.03	0.02	0.02
Spleen	10.29	2.92	3.08	0.78	2.24	0.52	2.62	1.01	1.21	0.42	0.85	0.19	0.42	0.47
Fat	1.60	0.47	1.27	0.72	0.34	0.26	0.51	0.67	0.27	0.36	0.56	0.17	0.33	0.35
Muscle	0.53	0.11	0.12	0.05	0.05	0.02	0.05	0.03	0.09	0.15	0.03	0.01	0.01	0.01
Small intestine	1.19	0.26	0.13	0.04	0.06	0.02	0.06	0.02	0.02	0.01	0.01	0.00	0.01	0.01
Large intestine	2.41	1.14	0.37	0.08	0.10	0.02	0.11	0.01	0.03	0.01	0.02	0.00	0.01	0.01
PIP	62.35	37.54	33.23	15.14	71.98	31.73	86.60	56.09	51.10	33.87	32.86	8.95	15.62	10.51
flu	1.40	0.62	0.26	0.13	0.20	0.15	0.25	0.27	0.11	0.18	0.04	0.03	0.04	0.07
PIP:flu	44		128		357		353		478		909		383	
PIP:kidney					1.9		2.0		3.2		3.0		2.5	

*Kidney uptake at 1 hr and 24 hrs was not evaluated in this study. In previously published biodistribution study in flank tumor-bearing mice, kidney uptake was 121 +/- 17 %ID/g at 1 hr and 234 +/- 140 %ID/g at 24 hrs [1].

Abbreviation: SD: standard deviation

Table S3: Long-term biodistribution of ^{125}I-DCIBzL in normal tissues

	% Injected Dose/gram											
	2 weeks		4 weeks		6 weeks		8 weeks		10 weeks		12 weeks	
	Mean	SD	Mean	SD	Mean	SD	Mean	SD	Mean	SD	Mean	SD
Blood	0.01	0.00	0.00	0.00	0.00	0.00	0.00	0.00	0.00	0.00	0.00	0.00
Salivary gland	0.13	0.10	0.05	0.02	0.02	0.02	0.01	0.01	0.00	0.00	0.00	0.00
Lung	0.31	0.26	0.12	0.04	0.05	0.05	0.03	0.04	0.00	0.01	0.01	0.01
Heart	0.05	0.04	0.02	0.01	0.01	0.00	0.00	0.00	0.00	0.00	0.00	0.00
Liver	0.05	0.04	0.02	0.01	0.01	0.01	0.00	0.00	0.00	0.00	0.00	0.00
Kidney	12.82	10.98	4.65	1.45	1.73	1.33	0.86	0.72	0.10	0.14	0.20	0.15
Bladder	0.15	0.11	0.06	0.02	0.04	0.05	0.01	0.01	0.00	0.01	0.00	0.00
Stomach	0.05	0.05	0.02	0.01	0.01	0.01	0.00	0.00	0.00	0.00	0.00	0.00
Pancreas	0.07	0.05	0.03	0.01	0.02	0.03	0.01	0.01	0.00	0.00	0.00	0.00
Spleen	0.77	0.59	0.36	0.16	0.10	0.08	0.05	0.04	0.01	0.02	0.01	0.01
Fat	0.32	0.25	0.12	0.05	0.05	0.07	0.02	0.02	0.01	0.01	0.01	0.00
Muscle	0.05	0.06	0.03	0.01	0.02	0.01	0.01	0.01	0.00	0.00	0.00	0.00
Small intestine	0.03	0.03	0.02	0.01	0.01	0.02	0.01	0.01	0.00	0.00	0.00	0.00
Large intestine	0.05	0.03	0.02	0.01	0.02	0.04	0.01	0.00	0.00	0.00	0.00	0.00

Abbreviation: SD: standard deviation

Reference

1. Chen Y, Foss CA, Byun Y, et al. Radiohalogenated Prostate-Specific Membrane Antigen (PSMA)-Based Ureas as Imaging Agents for Prostate Cancer. J Med Chem. 2008; 51: 7933–43.
